# Contribution of Corneal Neovascularization to Dendritic Cell Migration into the Central Area during Human Corneal Infection

**DOI:** 10.1371/journal.pone.0109859

**Published:** 2014-10-09

**Authors:** Mari Narumi, Yoshiko Kashiwagi, Hiroyuki Namba, Rintaro Ohe, Mitsunori Yamakawa, Hidetoshi Yamashita

**Affiliations:** 1 Department of Ophthalmology and Visual Sciences, Yamagata University Faculty of Medicine, Yamagata, Japan; 2 Department of Health and Nutrition, Yamagata Prefectural Yonezawa Women’s Junior College, Yamagata, Japan; 3 Department of Pathological Diagnostics, Yamagata University Faculty of Medicine, Yamagata, Japan; Cedars-Sinai Medical Center; UCLA School of Medicine, United States of America

## Abstract

Compared with the peripheral corneal limbus, the human central cornea lacks blood vessels, which is responsible for its immunologically privileged status and high transparency. Dendritic cells (DCs) are present in the central avascular area of inflamed corneas, but the mechanisms of their migration to this location are poorly understood. Here, we investigated the contribution of vessel formation to DC migration into the central cornea, and analyzed the DC chemotactic factors produced by human corneal epithelial (HCE) cells. Using human eyes obtained from surgical procedures, we then assessed vessel formation, DC distribution, and activin A expression immunohistochemically. The results demonstrated increased numbers of vessels and DCs in the central area of inflamed corneas, and a positive correlation between the number of vessels and DCs. Activin A was expressed in the subepithelial space and the endothelium of newly formed blood vessels in the inflamed cornea. In infected corneas, DCs were present in the central area but no vascularization was observed, suggesting the presence of chemotactic factors that induced DC migration from the limbal vessels. To test this hypothesis, we assessed the migration of monocyte-derived DCs toward HCE cell supernatants with or without lipopolysaccharide (LPS) stimulation of HCE cells and inflammatory cytokines (released by HCE cells). DCs migrated toward tumor necrosis factor alpha (TNF-α), interleukin (IL)-6, and activin A, as well as LPS-stimulated HCE cell supernatants. The supernatant contained elevated TNF-α, IL-6, and activin A levels, suggesting that they were produced by HCE cells after LPS stimulation. Therefore, vessels in the central cornea might constitute a DC migration route, and activin A expressed in the endothelium of newly formed vessels might contribute to corneal vascularization. Activin A also functions as a chemotactic factor, similar to HCE-produced TNF-α and IL-6. These findings enhance our understanding of the pathophysiology of corneal inflammation during infection.

## Introduction

Unlike most other organs, the central part of the human cornea lacks blood vessels and lymphatic vessels. This anatomical feature is necessary for high transparency and good visual acuity, and it contributes to its immunologically privileged status. As in other tissues, antigen-presenting cells (APCs) such as macrophages, Langerhans cells (LCs), and dendritic cells (DCs) are present in the human cornea, and participate in corneal immunity [1]–[3].

Hamrah and Dana [4] demonstrated that corneal LCs upregulate the expression of co-stimulatory molecules such as CD80 and CD86 in inflamed corneas. Mayer [5] described the characteristics of DCs in corneal buttons that were enucleated for transplantation purposes, and demonstrated the presence of LCs and immature DCs (imDCs) in the human corneal epithelium, and DC-SIGN-positive (i.e., CD209^+^) DCs in the stroma. These studies also reported that the number of APCs in the central part of the cornea was lower than that in the paracentral and peripheral regions.

In general, the recruitment of APCs in inflamed organs occurs through vessels, and the cells then migrate back to draining lymph nodes to accelerate the T-cell responses [6]. In terms of protecting the cornea from infection, its avascularity and small numbers of distributed APCs in the central part could be limiting factors. Prolonged inflammation often induces the formation of novel vessels in the central region of the cornea; however, this leads to a poor prognosis for visual acuity. A certain amount of APC recruitment and vessel formation is necessary to combat a corneal infection. Thus, understanding both the pathophysiology of APC movements in the cornea and their relationship with vessel formation might help identify therapeutic targets for regulating the corneal inflammatory response to infection.

In this study, we characterized DCs in the human cornea using infected and uninfected corneal tissues obtained from surgical units. We first analyzed the relationship between the distribution of DCs and the newly formed vessels, and found that the number of DCs in the central cornea increased during infection and/or vessel formation. In addition, DCs were detected in the central cornea in the absence of vascularization in some infected samples. Accordingly, we performed additional experiments with chemotactic factors that induce the migration of DCs into the central part of the cornea, and found that interleukin 6 (IL-6), tumor necrosis factor alpha (TNF-α), and activin A, which are produced by corneal epithelial cells and/or DCs, are involved in DC migration.

## Materials and Methods

### Materials

This study was performed in compliance with the tenets of the Declaration of Helsinki. All experiments were performed after approval from the Ethical Committee of Yamagata University Faculty of Medicine. After securing written informed consent from the subjects, we obtained corneal tissues surgically. The sample tissues in this study consisted of 28 eyes from 27 patients (13 males and 14 females) aged between 34 and 94 years (mean, 75 years). Six eyes were obtained by enucleation, and 22 corneal buttons were obtained during penetrating keratoplasty (PKP). The cases included seven infectious corneal ulcers and/or corneal perforation, six corneal degenerative diseases, six graft failures due to endothelial dysfunction, five herpetic keratitis infections, two choroidal malignant melanomas, one corneal perforation due to an autoimmune ulcer, and one corneal perforation from trauma. The precise sample data are shown in Table 1. Any cases with a history of herpetic keratitis were excluded from the cases of graft failure because of endothelial dysfunction. All five cases of herpetic keratitis showed typical epithelial keratitis (dendritic) when treatment was started. These cases included two corneal perforations. Cases 24 and 25 were tissues from the same patients as previous samples because of the recurrence of herpetic keratitis after PKP.

**Table 1:** Number of dendritic cells (DCs) and vessels in the central cornea.

Case	Age	Sex	Cause	Culture	Operation	CD1a	Langerin	DC-SIGN	CD83	Lymphatic vessels	Blood vessels
1	78	F	Malignalt choroidal melanoma	N.P	enucleation	N.D	N.D	N.D	N.D	N.D	N.D
2	37	M	Malignat choroidal melanoma	N.P	enucleation	N.D	0.34	N.D	N.D	N.D	N.D
3	64	M	Bullous keratopathy	N.P	PKP	1.05	0.53	0.53	1.05	N.D	1.05
4	34	M	Keratoconus	N.P	PKP	N.D	N.D	N.D	N.D	N.D	N.D
5	77	F	Bullous keratopathy	N.P	PKP	0.25	N.D	0.12	N.D	N.D	0.86
6	74	F	Bullous keratopathy	N.P	PKP	N.D	0.52	N.D	N.D	0.26	N.D
7	68	F	Bullous keratopathy	N.P	PKP	N.D	N.D	N.D	N.D	N.D	N.D
8	90	F	Bullous keratopathy	N.P	PKP	N.D	N.D	N.D	N.D	0.45	0.56
9	63	M	Trauma,	N.P	PKP	N.D	N.D	N.D	N.D	2.45	2.45
			Corneal perforation								
10	78	F	Graft failure	N.P	PKP	N.D	N.D	0.3	N.D	N.D	N.D
11	78	F	Graft failure	N.P	PKP	0.78	0.39	N.D	0.39	0.98	7.23
12	75	F	Graft failure	N.P	PKP	0.18	0.18	N.D	N.D	0.18	N.D
13	70	M	Graft failure	N.P	PKP	2.58	3.23	N.D	3.23	2.58	2.58
14	79	F	Graft failure	N.P	PKP	0.65	N.D	N.D	N.D	N.D	N.D
15	58	M	Graft failure	N.P	PKP	N.D	N.D	0.31	N.D	N.D	N.D
16	92	F	Bacterial keratitis	N.D	enucleation	N.D	0.42	0.42	N.D	1.68	N.D
			Corneal perforation								
17	64	M	Bacterial endophthalmitis,	N.D	PKP	6.83	1.25	4.21	2.62	3.08	9.57
			Bacterial keratitis								
18	94	F	Bacterial corneal ulcer	N.D	PKP	1.31	0.49	18.23	17.73	0.66	3.61
19	85	F	Bacterial endophthalmitis	P. aeurginosa	enucleation	0.33	0.5	4.33	0.5	2.5	7.67
20	78	F	Bacterial keratitis, Corneal perforation, Autoimmune ulcer	N.D	PKP	0.3	0.3	0.3	0.3	2.11	N.D
21	72	M	Fungal keratitis,	filamentous fungi	PKP	0.14	0.29	0.29	0.29	0.14	N.D
			Corneal perforation								
22	69	M	Trauma, Cornel perforation,	N.D	enucleation	0.91	0.54	2.36	0.18	1.99	1.45
			Post PKP, Bacterial infection								
23	76	M	Fungal keratitis,	filamentous fungi	PKP	0.38	0.19	1.5	2.63	0.75	N.D
			Corneal perforation								
24	67	M	Herpetic keratitis, Corneal perforation	N.P	PKP	0.00	0.44	2.22	0.59	2.63	0.00
25	70	M	Post PKP, Herpetic keratitis	N.P	PKP	3.01	0.91	0.78	0.00	0.00	24.31
26	68	F	Herpetic keratitis	N.P	PKP	2.58	0.86	3.23	0.00	0.00	2.58
27	59	M	Herpetic keratitis	N.P	PKP	0.00	0.00	0.00	0.00	0.00	5.61
28	72	M	Herpetic keratitis, Graft failure	N.P	PKP	0	0	0.31	0	0	0

M, male; F, female; N.P., not performed; N.D., not detected; PKP, penetrating keratoplasty.

Seven cases were defined as infected because of clinical findings that supported corneal infection, including focus in the cornea (7/7) and the presence of hypopyon (5/7), in slit-lamp examinations. The infection-causing pathogens were confirmed by cultures in three out of the seven infectious corneal ulcer and/or corneal perforation cases. The patient with autoimmune disease had corneal perforation 1 week after cataract surgery. Although the location of the ulcer was the epicenter, a slit-lamp examination showed the formation of a focus. This patient had discharge, hypopyon, and injection of the conjunctiva, which also strongly suggested the presence of infection. The infiltration of neutrophils in the subepithelial area and stroma of the cornea was confirmed histopathologically in all infected samples, as well as by reactivity to antibiotics and antifungal treatments.

### Immunohistochemistry (IHC)

IHC analyses were performed using paraffin-embedded corneal tissues and primary antibodies against CD1a (MTB1; mouse IgG1, κ; 1∶30 dilution; Novocastra; Newcastle-upon-Tyne, UK), DC-SIGN (CD209; H-200; rabbit polyclonal; 1∶400; Santa Cruz Biotechnology; Santa Cruz, CA, USA), langerin (CD207; 12D6; mouse IgG2b; 1∶100; Ylem S.R.L.; Rome, Italy), and CD83 (1H4b; mouse IgG1, κ; 1∶40; Novocastra). The expression of MHC-class II molecules on DCs was analyzed using anti-s-100 (rabbit polyclonal; ready to use; Nichirei, Tokyo, Japan) and anti-MHC-class II antibodies (mouse IgG2a; 1∶80; Abcam; Cambridge, UK). Corneal blood vessel formation was analyzed using anti-CD31 antibodies (JC70A; mouse IgG1, κ; 1∶50; Abcam), and anti-von Willebrand factor (factor VIII (rabbit polyclonal; 1∶1000, Dako; Carpinteria, California, USA). Lymphatic vessel formation was studied using anti-D2-40 (mouse IgG1, κ, 1∶50, Dako) and anti-vascular endothelium growth factor receptor-3 (VEGFR-3) antibodies (D1–D7; rabbit polyclonal; 1∶40; Abcam). VEGFR-3 is the specific receptor for VEGF-C and VEGF-D, which induce lymphangiogenesis [7], [8]. The presence of activin A, a chemotactic factor in DCs [9], [10], was confirmed using anti-activin A antibodies (goat polyclonal; 1∶50; R&D Systems; Minneapolis, MN, USA).

Formalin-fixed, paraffin-embedded (FFPE) tissues were sliced into 3-µm-thick sections. The sections were then deparaffinized, and endogenous peroxidase activity was blocked using methanol containing 0.3% hydrogen peroxide. Antigen retrieval was performed using ethylenediamine tetraacetic acid (antigen retrieval solution, pH 9; Nichirei Biosciences; Tokyo, Japan) in an autoclave (2 atm, 121°C, 20 min). After washing in phosphate-buffered saline (0.01 M, pH 7.4), tissue sections were incubated with primary antibodies at room temperature overnight. The labeled streptavidin-biotin peroxidase method (Ultra Tech HRP Streptavidin-Biotin Detection system, PN IM2391; Immunotech; Marseille, France) was used. Positive reactions were detected as brown coloration after incubation with 3,3′-diaminobenzidine tetrahydrochloride. The sections were counterstained with hematoxylin, and then observed using light microscopy.

### Multiple Immunofluorescence Staining

Multiple immunofluorescence staining of FFPE tissue sections was carried out as described previously [11]. Antigen retrieval was performed using the same method as described above in the IHC section. A cocktail of primary antibodies was applied to the tissue sections, which were incubated at room temperature overnight. A fluorescein-conjugated AffiniPure goat anti-rabbit IgG (H+L) antibody (Jackson ImmunoResearch Laboratories; West Grove, PA, USA) and a rhodamine-conjugated AffiniPure donkey anti-mouse IgG (H+L) antibody (Jackson ImmunoResearch Laboratories) were used as secondary antibodies, and the samples were mounted using Fluoromount (Diagnostic Biosystems Inc.; Pleasanton, CA, USA). Staining was observed using a fluorescent microscope.

### Evaluation of IHC Staining

The distribution of DCs and vessels in the central area of the corneas was evaluated. A NanoZoomer (Hamamatsu Photonics; Hamamatsu, Japan) was used to determine the central area and evaluate the size of the cornea. The type and density (per mm^2^ of a section) of infiltrating APCs, the number of blood and lymphatic vessels, and the expression of activin A in the corneal buttons (6–7 mm in diameter) obtained from PKP were observed using IHC. The anatomical limbus and the central area were determined in enucleated eyes. Cell counting was performed five times by two blinded observers, and the mean numbers were used.

### Isolation of Dendritic Cells

Peripheral blood samples were collected from healthy donors after securing written permission from the subjects. Peripheral blood mononuclear cells were isolated using Ficoll-Paque PLUS (GE Healthcare Life Science; Little Chalfont, Buckinghamshire, UK). After removing T and B cells using a DynaMag-15 Magnet with pan-T (CD2) and pan-B Dynabeads (CD19; Veritas; Tokyo, Japan), the eluted monocytes (1.0×10^6^ cells/well in 24-well tissue culture plates) were incubated in RPMI1640 medium supplemented with 10% fetal bovine serum, 50 ng/mL GM-CSF, and 20 ng/mL IL-4 (Primmune Inc.; Kobe, Japan) at 37°C and in a humidified incubator with 5.0% CO_2_, according to previously reported methods [12].

Monocyte-derived imDCs were harvested after 3–5 days of culture. After 3 days in culture, imDCs were stimulated with 1.5-µg/mL lipopolysaccharide (LPS; derived from *Escherichia coli* serotype O-157: B8; Sigma-Aldrich; St. Louis, MO, USA) to obtain mature DCs (mDCs). The purity of the population of isolated DCs was determined using immunocytochemistry.

Immunocytochemical analyses were performed using antibodies against CD1a, DC-SIGN, CD83, CD40 (11E9; mouse IgG2b; dilution titer, 1∶25; Novocastra), CD14 (7; mouse IgG2a; dilution titer, 1∶50; Novocastra), and MHC-class II (the same antibodies described above in the IHC section). The slides were fixed in 10% formalin for 10 min. Endogenous peroxidase activity was then blocked with methanol containing 0.3% hydrogen peroxide. Samples were incubated with primary antibodies overnight at room temperature. The method used for immunocytochemistry was the same as that described above for the IHC method. Approximately 80% of the imDCs used in this study expressed DC-SIGN, CD1a, and CD14, and approximately 90% expressed MHC-class II; however, only ∼10% expressed CD83 and CD40. Approximately 80% of the mDCs expressed CD83 and CD40, but only ∼10% expressed DC-SIGN, CD1a, and CD14.

### Culture of HCE Cells and Sampling of Supernatants

HCE cells (Ocucell; Corneal epithelial cells, Kurabo; Osaka, Japan) were cultured in a 28-cm^2^ dish according to the product manual. HCE cells at passage (P)3 or P4 were used in all experiments. Supernatants were collected from four treatment groups: (A) HCE cells, (B) HCE cells stimulated with 5 µg/mL LPS, (C) HCE cells co-cultured with imDCs (10^5^/well), and (D) HCE cells co-cultured with imDCs and stimulated with LPS. The co-culture of HCE cells and DCs was performed as described previously [13]. All samples were collected after centrifuging at 220×*g* for 10 min to remove cells, and were stored at −80°C for a maximum of 1 month.

### Migration Assays

DCs isolated from healthy donors (N = 5) were used in the migration assays. The migration of imDCs and mDCs was quantified using the Boyden chamber method. Migration assays were performed to assess for the movement of imDCs and mDCs into the supernatants of HCE cells from groups A and B described above. Migration assays were also performed in the presence of the following inflammatory cytokines: TNF-α, TGF-β_1_, IL-6, and activin A (R&D Systems), as described previously [14]–[16]. ImDCs and mDCs (2.0–3.0×10^5^/mL, 30 µL) were placed in the upper chamber, and supernatants A and B and the cytokine preparations were placed in the lower chamber. Membranes with 8-µm pores were used to trap the migrating DCs. The assays were performed in a humidified incubator at 37°C with 5% CO_2_ for 2 h. After incubation, the membranes were gently removed, and the migrating cells were visualized using Giemsa staining. The migration assays were repeated three times. The number of migrating cells was counted five times by one observer, and the mean numbers were calculated.

### Suppression of DC Migration by Neutralization with Anti-Activin A Antibodies

DCs isolated from healthy donors (N = 5) were used in migration assays. HCE cells were incubated with 1 µg/mL anti-activin A antibody (goat polyclonal; R&D Systems) for 12 h. The HCE cells were then stimulated with 5 µg/mL LPS, and supernatants (group E) were collected. Supernatants from group B HCE cells stimulated with 5 µg/mL LPS were also collected as a control. The migration of imDCs toward these supernatants was quantified using a CytoSelect^TM^96-well Cell Migration kit (Cell Biolabs, Inc.; San Diego, CA, USA) and the experiment was repeated three times.

### Enzyme-Linked Immunosorbent Assay (ELISA) in HCE Cell Supernatants

The concentrations of TNF-α, TGF-β_1_, IL-6, and activin A in HCE cell supernatants were quantified using ELISA kits (R&D Systems). The following supernatants were analyzed: (A) HCE cells, (B) HCE cells stimulated with LPS, (C) HCE cells co-cultured with imDCs, and (D) HCE cells co-cultured with imDCs and stimulated with LPS. ImDCs were isolated from the peripheral blood of healthy donors (N = 7). Cells were incubated in a 28-cm^2^ dish for 1.5 h, 3 h, or 6 h at 37°C in an incubator with 5% CO_2_ and a humidified atmosphere. The ELISAs were repeated four times to ensure consistent results.

### Statistical Analysis

Statistical analyses were performed using SPSS ver. 18.00 (IBM; Chicago, IL, USA). The numbers of DCs and vessels, and expression of activin A were analyzed using Mann-Whitney U tests with Spearman’s rank correlation coefficients. The cytokine levels in supernatants were analyzed using analysis of variance (ANOVA) with Bonferroni correction. The numbers of DCs in the migration assays were analyzed using Mann-Whitney U tests. Differences with *p*<0.05 were considered statistically significant. In addition, stepwise multiple regression analysis was used to analyze the factors relevant to the distribution of DCs (Table 2). The independent variable was the number of DCs, and the dependent variables included regraft, the number of blood and lymphatic vessels, and the presence of infection.

**Table 2:** Numbers of dendritic cells and vessels (per mm^2^) in the central area of the cornea.

Dendritic cells/vessels	Non-infected (N = 15)	Infected (N = 7)	*P value* A)
**CD1a^+^ DCs**	0.37	1.28	0.06
**Langerin^+^ DCs**	0.35	0.50	0.02*
**DC-SIGN^+^ DCs**	0.08	3.96	0.00*
**CD83^+^ DCs**	0.31	3.03	0.01*
**Lymphatic vessels**	0.46	1.61	0.01*
**Blood vessels**	0.98	2.79	0.32

A) Mann-Whitney U test,

*p<0.05.

There were statistically significant differences in the numbers of langerin^+^ DCs, DC-SIGN^+^ DCs, CD83^+^ DCs, and lymphatic vessels between infected and non-infected cases. Herpetic keratitis cases were excluded from the statistical analyses. This is because they were all in chronic states without neutrophil infiltration; therefore, infections could not be defined.

## Results

### IHC

#### Distribution of DCs and vascularization of the cornea

**Figure 1:**
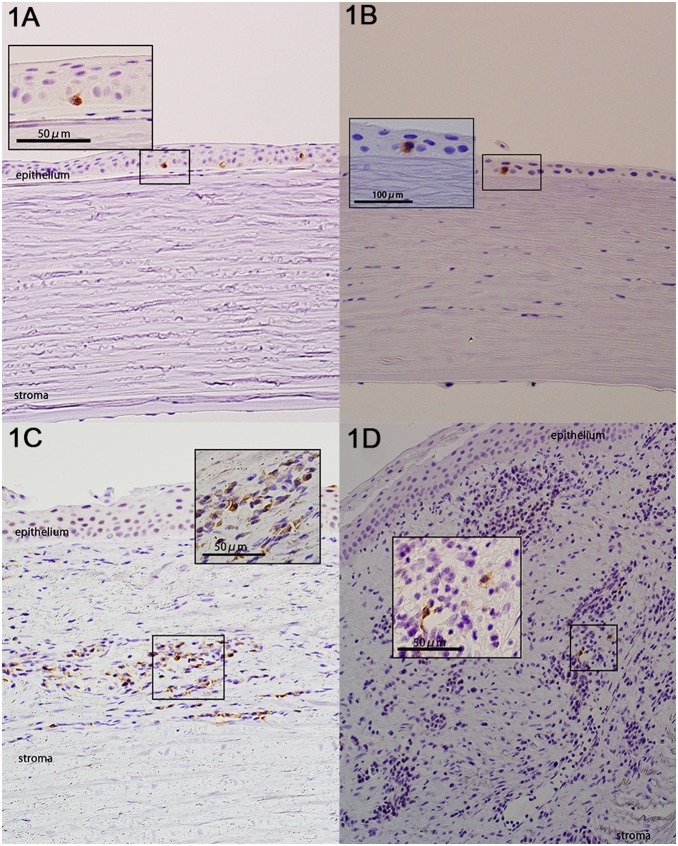
Distribution of dendritic cells (DCs). DCs were observed under a light microscope. Primary antibodies against CD1a (MTB1; mouse IgG1, κ; 1∶30 dilution; Novocastra; Newcastle-upon-Tyne, UK), DC-SIGN (CD209; H-200; rabbit polyclonal; 1∶400; Santa Cruz Biotechnology; Santa Cruz, CA, USA), langerin (CD207; 12D6; mouse IgG2b; 1∶100; Ylem S.R.L.; Rome, Italy), and CD83 (1H4b; mouse IgG1, κ; 1∶40; Novocastra) were used to observe DCs. **A.** Case 17; infected endophthalmitis. CD1a^+^ DCs were observed mainly in the epithelium and the subepithelial space of an inflamed cornea. **B.** Case 17; infected endophthalmitis. Langerin^+^ DCs were observed in the epithelium. **C.** Case 18; infected keratitis. DC-SIGN^+^ DCs were observed in the stroma. **D.** Case 22; corneal perforation, post PKP. CD83^+^ DCs were observed in the subepithelial space together with lymphocytic infiltration.

The infiltration of inflammatory cells, including neutrophils, was observed in seven cases of infectious corneal ulcer or corneal perforations. CD1a^+^ DCs were observed mainly in the epithelium and the subepithelial space. Langerin^+^ DCs (LCs) were observed in the epithelium. DC-SIGN^+^ DCs were observed in the subepithelial space and in the stroma. CD83^+^ DCs were found in the subepithelial space and in the upper layers of the stroma. Each type of DC was present simultaneously with inflammatory cells (Fig. 1). In inflamed corneas, the mean percentage of expression of MHC-class II molecules on the DCs was 64.3%, compared with 75.0% in non-inflamed corneas.

**Figure 2:**
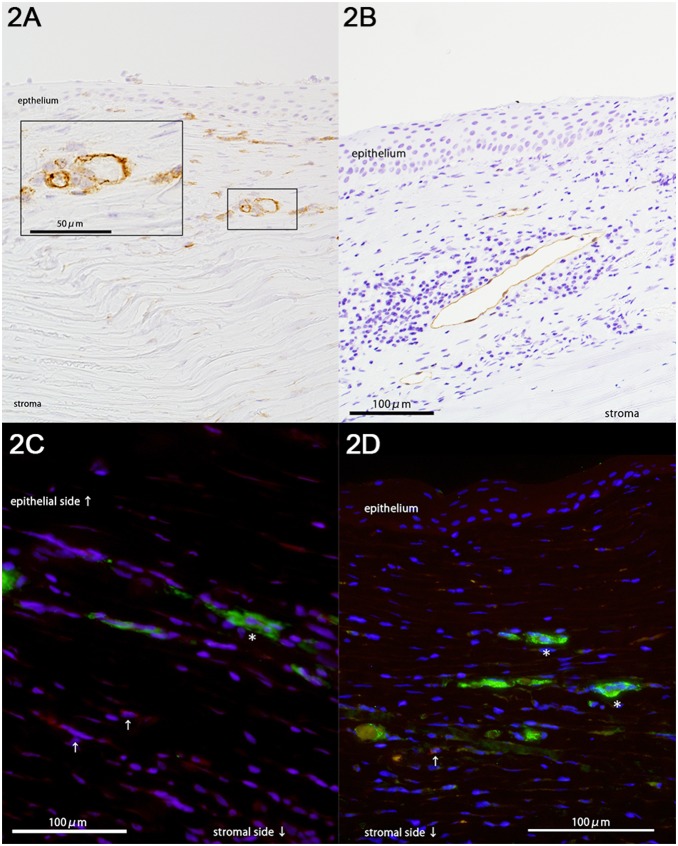
Vessel formations and DCs. **A.** Case 18; infected keratitis. Blood vessel formation was observed in the upper stroma. Blood vessels were determined by staining with anti-CD31 antibodies (JC70A; mouse IgG1,κ; 1∶50; Abcam). **B.** Case 17; infected endophthalmitis. Lymphatic vessel formation was assessed using anti-D2-40 antibodies (mouse IgG1, κ, 1∶50, Dako). Lymphatic vessel formation was observed in the subepithelial space. **C.** Case 18; infected keratitis. Lymphatic vessel was determined using anti-D2-40 (mouse IgG1, κ, 1∶50, Dako) antibodies, and immature DCs were identified using anti-DC-SIGN antibodies (CD209; H-200; rabbit polyclonal; 1∶400; Santa Cruz Biotechnology; Santa Cruz, CA, USA). A merged image of immunofluorescence staining for D2-40 (green), DC-SIGN (red), and DAPI (blue) captured using a fluorescence microscope. DC-SIGN^+^ DCs (arrows) were observed around a lymphatic vessel (*). **D.** Case 17; infected endophthalmitis. Blood vessels were determined using anti-von Willebrand factor staining (factor VIII (rabbit polyclonal; 1∶1000, Dako; Carpinteria, California, USA), and mature DCs were identified using anti-CD83 antibodies (1H4b; mouse IgG1, κ; 1∶40; Novocastra). A merged image of immunofluorescence staining for factor 8 (green), CD83 (red), and DAPI (blue) was captured using a fluorescence microscope. CD83^+^ DCs (arrow) were observed around the blood vessels (*).

**Figure 3:**
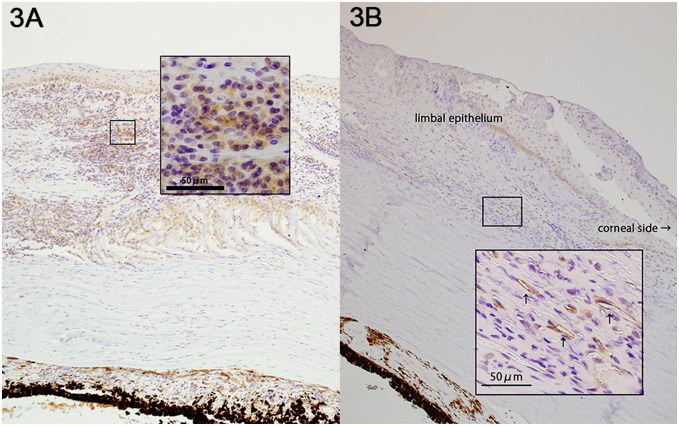
Expression of activin A. Activin A expression was determined using anti-activin A antibodies (goat polyclonal; 1∶50; R&D Systems; Minneapolis, MN, USA). **A.** Case 17; infected endophthalmitis. Activin A was expressed in the corneal epithelial and the subepithelial space with inflammatory cells. **B.** Case 19; bacterial endophthalmitis. Activin A was expressed in the endothelium of newly formed blood vessels, which suggests the presence of vessel formation in the central cornea.

Blood vessels, lymphatic vessels, and corneal inflammation were more abundant in cases of infection and graft failure than choroidal tumors and degenerative diseases. Blood vessels were found in the stroma and the subepithelial space. Lymphatic vessels were mainly present in the subepithelial space (Fig. 2). VEGF-R3 was observed in the endothelium of the lymphatic vessels, and DCs were observed around these newly formed vessels (Fig. 3).

#### Correlations between DCs and vessels

The numbers of DCs were compared between the infected and uninfected cases. The five cases with herpetic keratitis were excluded from the present statistical analysis because clinical and histopathological evaluation revealed that the corneas were in a chronic state. Neutrophil infiltration was not detected and the presence of infection could not be confirmed in these cases. The numbers of langerin^+^ DCs, DC-SIGN^+^ DCs, and CD83^+^ DCs were significantly greater in the cases with infection compared with those without infection (Table 2).

There were statistically significant correlations between the numbers of blood vessels and lymphatic vessels (*p* = 0.002; Spearman’s rank correlation coefficient). There were also statistically significant correlations between the numbers of blood vessels and DCs expressing CD1a (*p* = 0.001), langerin (*p* = 0.01), and CD83 (*p* = 0.002; Spearman’s rank correlation coefficient). Additional statistically significant correlations were observed between the numbers of lymphatic vessels and of DCs expressing CD1a (*p* = 0.023), langerin (*p* = 0.002), and CD83 (*p* = 0.004; Spearman’s rank correlation coefficient). Large numbers of lymphatic vessels were observed in cases of infection (*p* = 0.01), but there was no significant difference in the numbers of blood vessels between the infected and the non-infected cases (Mann-Whitney *U* test, *p* = 0.318; Table 2).

#### Expression of activin A

Activin A was detected in the cytoplasm of epithelial cells in both the infected and the non-infected corneas. Activin A in the subepithelial space and stroma was observed in all the cases of infection, but not in the cases without infection. Inflammatory cells and DCs were simultaneously present along with activin A in the subepithelial space (Fig. 3A, B). In two of the seven infected cases (cases 19 and 22), activin A was detected in the endothelium of blood vessels (Fig. 3B).

Activin A expression in the subepithelial space (N = 8), large numbers of DCs expressing CD1a (*p* = 0.002), DC-SIGN (*p* = 0.002), and CD83 (*p* = 0.007, Mann-Whitney *U* test) were detected. There was no significant difference in the numbers of blood vessels and lymphatic vessels between cases with and without the expression of activin A in the subepithelial space.

### Multiple Regression Analysis

Data revealed an increased number of DC-SIGN^+^ imDCs and CD83^+^ mDCs in the infected cases. The large number of CD1a^+^ imDCs was related to the increase in the number of blood vessels, whereas the large number of langerin^+^ DCs was related to the increase in the formation of lymphatic vessels. The regraft status was not related either to the increased numbers of any specific type of DC or to the increased number of blood vessels and/or lymphatic vessels (Table 3).

**Table 3:** Multiple regression analysis.

Dependent variable	Independent variable	Regression coefficient β	*P value* A)
DC-SIGN (immature DCs)	Infection	0.537	0.01*
CD1a (immature DCs)	Blood vessels	0.683	0.00*
CD83 (mature DCs)	Infection	0.471	0.02*
Langerin (Langerhans cells)	Lymphatic vessels	0.557	0.00*

A) Mann-Whitney U test,

*p<0.05.

### Migration Assay

ImDCs migrated toward LPS-stimulated HCE cell supernatant (group B), but not toward that from HCE cells only (group A), whereas few mDCs migrated toward either supernatant. ImDCs, but few mDCs, also migrated toward all preparations of TNF-α, TGF-β_1_, IL-6, and activin A (Table 4).

**Table 4:** Numbers of dendritic cells (DCs) that migrated toward the supernatants of human corneal epithelial cells that were either stimulated or not stimulated with lipopolysaccharide (LPS).

	N	Immature DCs	Mature DCs	*P value* A)
**LPS (−)**	7	2.6	3.6	1.000
**LPS (+)**	7	106.1	3.3	0.00*
***P value*** A)		0.00*	0.45	

A) Mann-Whitney U test,

*p<0.05.

The numbers of migratory dendritic cells in each well of the Boyden chamber are shown. LPS (−), supernatants from human corneal epithelial cells without LPS stimulation. LPS (+), supernatants from human corneal epithelial cells stimulated with LPS for 1.5 hours.

### Suppression of imDC Migration by anti-Activin A

The migration of ImDCs for 1.5 h toward LPS-stimulated HCE cell supernatants with (group E) or without (group B) neutralization with anti-activin A antibodies was evaluated using relative fluorescent units (RFU). Neutralization with anti-activin A antibodies reduced the migration of imDCs toward LPS-stimulated HCE cell supernatants by ∼40%. However, there was no statistically significant difference between the two treatment groups (group B [N = 19], 7.49 RFU vs. group E [N = 30], 4.39 (RFU); Mann-Whitney U test, *p* = 0.850].

### ELISA

**Figure 4:**
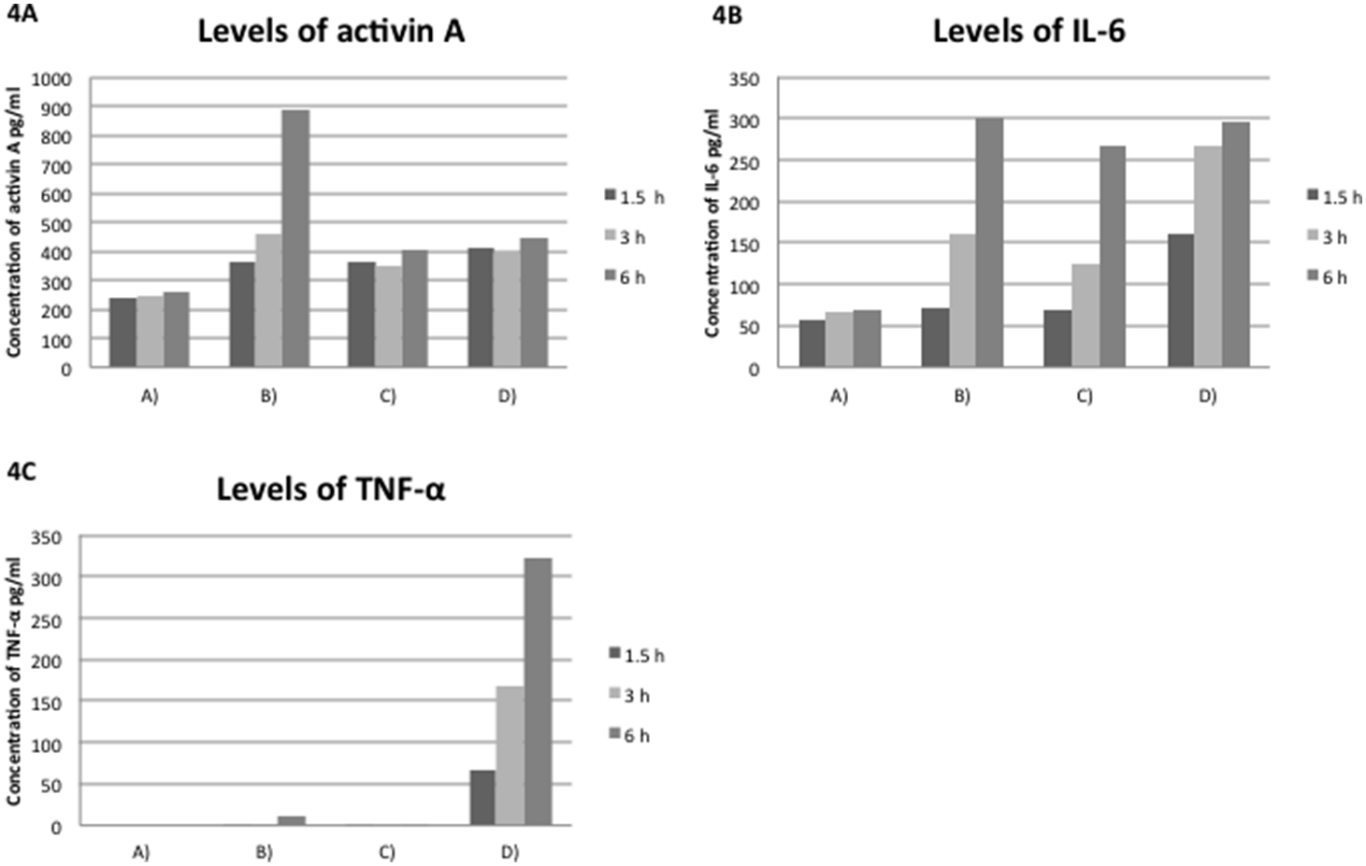
Increasing levels of cytokines in the supernatant of human corneal epithelial (HCE) cells. **A.** Levels of activin A in the supernatants from group A (HCE cells), B (HCE cells stimulated with 5 µg/mL LPS), C (HCE cells co-cultured with 10^5^/well, and D (HCE cells co-cultured with imDCs and stimulated with LPS for 1 hour, 3 hours, and 6 hours). The levels of activin A increased in a time-dependent manner in supernatants from group B. After 6 hours, they were higher in groups B and D than in group C (*p*<0.01, Uni-ANOVA with a Bonferroni correction). **B.** Levels of IL-6 in the supernatants of the groups described in **A.** The levels of IL-6 increased in a time-dependent manner in groups B, C, and D. The levels of IL-6 increased after 6 hours in supernatants from groups B, C, and D, but were undetectable in those from group A. There were no significant differences in the levels of IL-6 among the supernatants from groups B, C, and D after 6 hours. **C.** Levels of TNF-α in supernatants from the groups described in **A.** TNF-α was not detectable in those from group A. TNF-α levels increased significantly only in group D, and the increase was time-dependent (*p*<0.01, Uni-ANOVA with a Bonferroni correction).

TGF-β_1_ was not detected in any of the supernatant samples. The results of ELISAs for activin A, IL-6, and TNF-α are shown in Fig. 4. TNF-α was not detected in HCE cell supernatants from groups A, B, or C. In the supernatants from group D (LPS-stimulated HCE cells co-cultured with imDCs), elevated levels of TNF-α were observed in a time-dependent manner. IL-6 was detected in all supernatant samples. The elevation in IL-6 concentrations occurred in a time-dependent manner, except in supernatants from untreated HCE cells (group A). There were no significant differences in the IL-6 concentrations in the supernatants from groups B, C, or D after 6 h. Activin A was detected in all supernatants, but the increase in activin A concentrations was only time-dependent in HCE cells supernatants stimulated with LPS (group B).

## Discussion

This study revealed that an increased number of DCs that expressed CD1a, langerin, DC-SIGN, and CD83 were distributed in the central cornea in cases with acute inflammatory cell infiltration and vessel formation. DC-SIGN is also expressed on M2 macrophages. Therefore the DC-SIGN^+^ DCs that were observed in the stroma might include macrophages. Mayer et al. [5] revealed the presence of some types of DCs in the central human cornea, consistent with the observations in the current study. In addition, Yamagami et al. reported the presence of DCs in the corneal epithelium of human donors, which were MHC-class II positive and from the myeloid lineage [17]. Mastropasqua et al. showed the presence of DCs in normal and inflamed corneal epithelium using confocal microscopy [18], as well as an increased number of distributed DCs in inflammatory states. Although the study by Masropasqua et al. might have included both dendritic cells and macrophages because they defined DCs only using morphological findings, the results of these studies [5], [17]–[18] are supportive of the current observations. Therefore, it is possible that the DCs present in the human cornea could induce the recruitment of additional migrating DCs for protection.

The current study revealed that vessel formation contributed to the migration of DCs into the central cornea. Large numbers of vessels in the central area could potentially induce a much faster recruitment of DCs than could the peripheral limbal vessels. Vessel formation was observed in cases with infection, herpetic keratitis, and graft failure. Most cases of corneal perforation after trauma injury are emergencies, and vascularization is rarely observed in the cornea. However, in the current study vessel formation was observed in case 9, which exhibited corneal perforation due to trauma. This is because the patient had a history of medically treated corneal perforation before the trauma episode.

The five cases of herpetic keratitis were diagnosed according to the guidelines for herpetic keratitis from the AAO PPP Committee (Secretary for Quality of Care, Hoskins Center for Quality Eye Care). These cases were excluded from the statistical analysis of the number of distributed DCs and vessels between infected and non-infected cases (Table 2). This is because clinical and histopathological evaluations revealed that they were in the chronic state, and no neutrophils were detected. In addition, corneal perforation occurred in two of the five cases, and another two cases had recurrence of herpes virus epithelial keratitis after PKP. Therefore, we determined that these complicated backgrounds might have affected the results. In all herpetic keratitis cases, increased numbers of DCs were observed in the central area. In three of these, the herpetic keratitis resulted in significant vessel formation that might have contributed to DC migration. However, there were no statistical correlations between any types of DC and blood vessels or lymphatic vessels. Nevertheless, a study analyzing large numbers of herpetic keratitis cases from different phases is necessary to clarify the distribution of DCs during viral infection.

Activin A is a diametric glycoprotein that belongs to the TGF-β superfamily. The activin family promotes the secretion of follicle-stimulating hormone (FSH). Many additional studies have revealed that activin A is a wide-ranging cytokine and proliferative factor that functions in the reproductive system, during DC chemotaxis [9]–[10], [19]–[20], and in developmental processes such as the developing nervous system [21]. In eyes, it plays roles in retinal development and inhibiting the proliferation of retinal pigment cells. In addition, the activin A receptor has been identified in the cornea (corneal epithelial cells, stromal cells, and endothelial cells), the epithelium of the ciliary body, in epithelial cells of the lens, and in retinal pigment cells [22]. However, the function of activin A in the human cornea has not yet been fully investigated. In the current study, activin A was expressed in the endothelium of newly formed blood vessels in infected corneas. Activin A promotes neovascularization in the cornea [23]; therefore, this observation suggests that activin A might promote blood vessel formation in infected and inflamed corneas. VEGFR-3 was expressed in the endothelium of the newly formed lymphatic vessels in the central part of infected corneas. It is a specific receptor for VEGF-C and VEGF-D, which induce lymphangiogenesis [7], [8]; therefore, the expression of VEGFR-3 is suggestive of the lymphatic vessel formation. As such, vessel formation in infected and inflamed corneas accelerates in the central area, and these vessels form an accelerated pathway for migratory DCs to enter the central cornea and protect it from infection.

IHC data in the current study revealed activin A expression in the subepithelial space of infected corneas (N = 7). In three of seven cases (cases 20, 21, and 23), the number of DCs increased significantly without blood vessel formation (Table 1). In addition, migration assays confirmed the migration of imDCs toward activin A and supernatants from LPS-stimulated HCE cells. ELISA also demonstrated constant production of activin A by HCE cells and DCs with or without stimulation by LPS. From these findings, we concluded that, at the early stage of infection without vessel formation in the central area, activin A induced the rapid recruitment of DCs into the central cornea from the peripheral limbal vessels. It is already known that the corneal limbus is vessel-rich, and that larger numbers of DCs are present there than in the central area [5], [17]–[18]. The recruitment of DCs from the limbus is another key pathway by which DCs enter the central cornea. Similar to the observations for activin A, our data also suggest the possible involvement of TNF-α and IL-6 in DC recruitment (Fig. 4). Although the production of cytokines by corneal cells has been studied widely [14]–[16] ours is the first report to demonstrate that some of these cytokines function as chemotactic factors to induce the migration of DCs into the central cornea. The results of migration assays in the presence of neutralizing anti-activin A antibodies revealed no statistically significant difference in the migration of imDCs toward the supernatants of HCE cells with or without stimulation with LPS. This suggests that activin A might be a chemotactic factor for DCs, but that it is not the only factor that stimulates the migration of DCs into the central cornea. As such, the function of activin A in the human cornea needs to be investigated further.

ELISA results in the current study showed that HCE cells stimulated with LPS produced IL-6, but not TNF-α; HCE cells only produced TNF-α when they were co-cultured with DCs and LPS. These results suggest that these two cytokines act at different stages of corneal infection. It also suggests that the secondary migration of DCs was induced by both the production of IL-6 by HCE cells during the early phase, which led to the recruitment of DCs into the central area, as well as the production of TNF-α as a result of the interaction between HCE cells and migratory DCs. Additional future studies would help clarify the mechanisms underlying the interactions between HCE cells and DCs.

Generally, dominant infiltration of neutrophils is a typical pathological finding in infected tissues. In this study it was very difficult to discuss the presence of infection based only on clinical findings because the detection rate of the pathogens was ∼40%. Therefore, we hypothesized that the use of topical antibiotics before the culture affected the detection rate. However, the rate was similar to that reported previously when corneal cultures were formed from 9934 patients [24]. Therefore to confirm infection we defined infected cases according to both clinical and pathological findings. For example, a corneal ulcer due to autoimmune disease is a non-infected ulcer. In the current study the patient with autoimmune disease (case 20) had corneal perforation 1 week after cataract surgery, and she was using topical steroid and antibiotics. She had symptoms of discharge and ocular pain. The slit lamp examination showed ulceration in the epicenter, hypopyon, injection of the conjunctiva, and focus formation, which together strongly suggested infection. However, a bacterial examination did not detect any causative pathogens. IHC revealed that the discharge was neutrophil-dominant and there was significant neutrophil infiltration into the ulcer; therefore, the patient was diagnosed with an infectious ulcer.

A limitation of this study is that we only used corneas from individuals with diseased states (Table 1); therefore, it is challenging to make rigorous comparisons of the numbers of DCs in these cases. Nevertheless, it was clear that fewer DCs were observed in cases from degenerative diseases or choroidal malignant melanomas, and no inflammatory cells were detected in the central cornea of these cases. In samples from patients with choroidal tumors, the cornea was intact, and the expression of activin A and the distribution of DCs in these cases were used as the control cases. Another limitation is that the subjects included those with a variety of diseases, past histories, and treatments. Therefore, not only infection, but also patients’ backgrounds might have affected the distribution of DCs in the cornea. Multiple regression analyses suggested that the number of DC-SIGN^+^ and CD83^+^ DCs increased with inflammatory cell infiltration, and that CD1a^+^ and langerin^+^ increased with vessel formation. It is likely that these are important for the recruitment of DCs.

## Conclusion

In inflamed human corneas, the number of migratory DCs increased in the avascular central area, probably to protect against infection. Newly formed corneal blood vessels and lymphatic vessels in the central cornea constitute faster paths for the migration of DCs into the central area. Activin A accelerates blood vessel formation and functions as a chemotactic factor for DCs in infected corneas. Similar to the inflammatory cytokines IL-6 and TNF-α, activin A (produced by HCE cells and imDCs) participates in the recruitment of DCs from the peripheral vessels. Therefore, the recruitment of DCs into the central cornea is orchestrated by vessel formation and the production of chemotactic factors from epithelial cells and inflammatory cells. Understanding the mechanism by which DCs migrate into the cornea will lead to the identification of therapeutic targets for corneal inflammation during corneal infection.
